# Camelid husbandry in the Atacama Desert? A stable isotope study of camelid bone collagen and textiles from the Lluta and Camarones Valleys, northern Chile

**DOI:** 10.1371/journal.pone.0228332

**Published:** 2020-03-11

**Authors:** Paul Szpak, Daniela Valenzuela

**Affiliations:** 1 Department of Anthropology, Trent University, Peterborough, Ontario, Canada; 2 Departamento de Antropología, Universidad de Tarapacá, Arica, Chile; Griffith University, AUSTRALIA

## Abstract

Management of camelids in the coastal valleys of the Andes has generated much debate in recent years. Zooarchaeological and isotopic studies have demonstrated that in the coastal valleys of northern and southern Peru there were locally maintained camelid herds. Because of the hyperarid conditions of the northern coast of Chile, this region has been assumed to be unsuitable for the raising of camelids. In this study we report stable carbon and nitrogen isotopic compositions of camelid bone collagen and textiles made from camelid fiber from Late Intermediate Period (LIP) and Late Horizon (LH) occupations in northern Chilean river valleys. The camelid bone collagen isotopic compositions are consistent with these animals originating in the highlands, although there is a significant difference in the camelids dating to the LIP and LH, possibly because of changes made to distribution and exchange networks by the Inca in the LH. There were no differences between the isotopic compositions of the camelid fibers sampled from textiles in the LIP and LH, suggesting that either the production of camelid fiber was unchanged by the Inca or the changes that were made do not present visible isotopic evidence. Several camelid fiber samples from both the LIP and LH present very high *δ*^13^C and *δ*^15^N values, comparable to human hair samples from one site (Huancarane) in the Camarones Valley. These data suggest that people in the northern valleys of Chile may have kept small numbers of animals specifically for fiber production. Overall, however, the vast majority of the textile samples have isotopic compositions that are consistent with an origin in the highlands. These data suggest that the hyperarid coastal river valleys of northern Chile did not support substantial camelid herds as has been interpreted for northern Peru.

## Introduction

Camelids (llama [*Lama glama*] and alpaca [*Vicugna pacos*]) were the only species of large animal to be domesticated in the Americas. Although they were not used for milk and traction, they were used extensively for their meat [1–3] and leather [4, 5], as beasts of burden in caravans [6, 7], and their dung was used for fertilizer and fuel [5, 8, 9]. Their most important economic role, however, was the production of fiber to be used to manufacture textiles. These textiles were of tremendous economic, social, and political importance throughout the Andean region [10, 11]. The Inca extracted labor taxes of rough cloth from households and also utilized fine cloth woven by high status, specialist weavers [12–14]. Under Inca control during the Late Horizon the *mit’a* was an institutionalized labor service through which conquered peoples paid tribute, providing an important source of income for the state [13, 15]. The *mit’a* consisted of a rotational labor system, including herding camelids, farming, and weaving, among other activities. The textile *mit’a* was one of the most important, since clothing was the object most valued by the Inca state in economic, political, and ritual terms [11, 13]. In the textile *mit’a* the state provided high quality fiber which was spun and woven into fine cloth, while women might have supplied their own tools [16].

Because textiles worn as clothing were highly visible, they were important embodiments of social identity, status, and power [17–19]. The way that scholars have thought about the role of camelids in Andean societies is strongly influenced by ethnographic and ethnohistoric descriptions, particularly those associated with the Inca. Based on these data, camelids have been seen as animals that reside in the high altitude grasslands of the *altiplano* (above 3,800 masl) and their presence in archaeological deposits outside of this narrow altitudinal range is sometimes assumed *a priori* to represent the presence of meat (dried as *charqui*) or fiber that was acquired via exchange with the highlands. Based on an extensive synthesis, Bonavia [20] concluded that there is little empirical evidence to support the notion that camelids are high altitude specialists, tracing the origins of this back to a statement made by Troll [21]. Bonavia [20] suggests that the natural habitat of the camelids “extends from sea level to altitudes over 5,000 masl, from the coastal deserts and fertile intermontane valleys to the high punas and the exuberant wet region of the *ceja de selva*, with all of the intermediate life zones that are far more varied than usually thought” (see also [22]).

Mounting isotopic evidence from the north coast of Peru has demonstrated the presence of local camelid populations in these areas from at least the Early Intermediate Period (c. 200 BC) [23–28]. Moreover, isotopic data demonstrate that camelids (probably llamas) were raised in an urban environment at the Wari site of Conchopata and intensively foddered with maize (>75% of the diet [29]). Shimada and Shimada [30] summarized other lines of evidence, which are also consistent with the raising of camelids on the north coast of Peru since at least the Middle Horizon (c. AD 600). Isotopic data indicate that meat and fiber was derived from local animals [26, 31, 32]. This finding runs counter to the widely-held assumption that the fiber in textiles found at low elevation sites must have originated in the highlands [19, 33], although in some cases (i.e., Chancay, central Peruvian coast, Late Intermediate Period) the fiber was exclusively imported from the highlands [31]. Nevertheless, these data are consistent with Bonavia’s conceptualization of camelids as highly adaptable to a wide variety of environments. What is still unclear, however, is just how widespread the occurrence of non-highland camelid husbandry was in the Andean region. The fact that these animals *could* survive in a given environment does not mean that they did.

The purpose of this study was to investigate if the inhabitants of two of the northern valleys of Chile (Lluta and Camarones) maintained local populations of camelids during the Late Intermediate Period and Late Horizon. Isotopic compositions of camelid skeletal remains suggest that these individuals most likely lived in the *altiplano*, although there were significant differences in the isotopic compositions of the LIP and LH camelid samples. The majority of fiber samples from textiles recovered from both LIP and LH contexts also originated in the *altiplano*, but a small percentage of the fibers from both periods were most likely derived from local animals, raised under very arid conditions at lower altitudes.

### Setting and archaeological context

The environment of our study area is one that is, in many ways, intermediate between southern Peru to the north and the central Atacama Desert to the south. The study area is located in the northern section of the Atacama Desert between 18° and 19°S [34]. Unlike the coast, which offers abundant and stable marine resources, the inland valleys of the northern Atacama Desert are comparatively unstable places for human occupation, due to low biological productivity, limited availability of fresh water, periodic droughts, and overall lower predictability of resources [35].

The Lluta and Camarones rivers originate in the highlands (Lluta at 3,900 masl and Camarones at 2,900 masl), draining into the Pacific Ocean. They are the only rivers in northernmost Chile that present permanent runoff throughout the year, but occasional prolonged droughts may cause their runoff to terminate in their lower courses [36]. These rivers are characterized by extremely high concentrations of metals (As, B, Cu, Li, Na, K) [37], which limits the quantity and quality of wild vegetation, restricting it to the riverbed and areas fed by spring water. In fact, the sites under study are strategically located in association with fresh water springs. The limited vegetation coverage and the quantity and quality of water available in the Lluta and Camarones valleys make them unsuitable for supporting large numbers of animals, both wild and domesticated. Pre-Hispanic populations inhabiting these valleys had access to a limited variety of plants, seeds, and fruits. Agriculture generated important complementary resources, although also limited in variety (possibly only maize in pre-Hispanic times).

According to regional paleoclimate records, since 3,000 BP the current hyper-arid conditions have predominated in the Atacama Desert, although there were significant increases in rainfall at various intervals including 1050–600 cal. BP − the Medieval Climatic Anomaly (MCA), which roughly coincides with the Late Intermediate Period and Late Horizon [38–40]. Although climatic conditions were similar to those at present, these rainy events would have been more frequent during the MCA and would have meant a recharge of the fluvial and spring water resources. It is presumed, therefore, that there was greater vegetation coverage and more water available during the periods under study than there is presently.

The Late Intermediate Period and Late Horizon witnessed the development of the Arica Culture as a group of segmented societies that shared a common cultural tradition, although they did not comprise a single political entity. These communities practiced a mixed agro-maritime subsistence and had differential settlement and architectural patterns, as well as interactions with other groups [41–43]. During the Late Horizon, the impact of the Inca in the region is demonstrated by multiple lines of evidence, including typical Inca-style goods, as well as other proxies for changes in local economic activities, settlement patterns, and health conditions [44–46].

**Fig 1:**
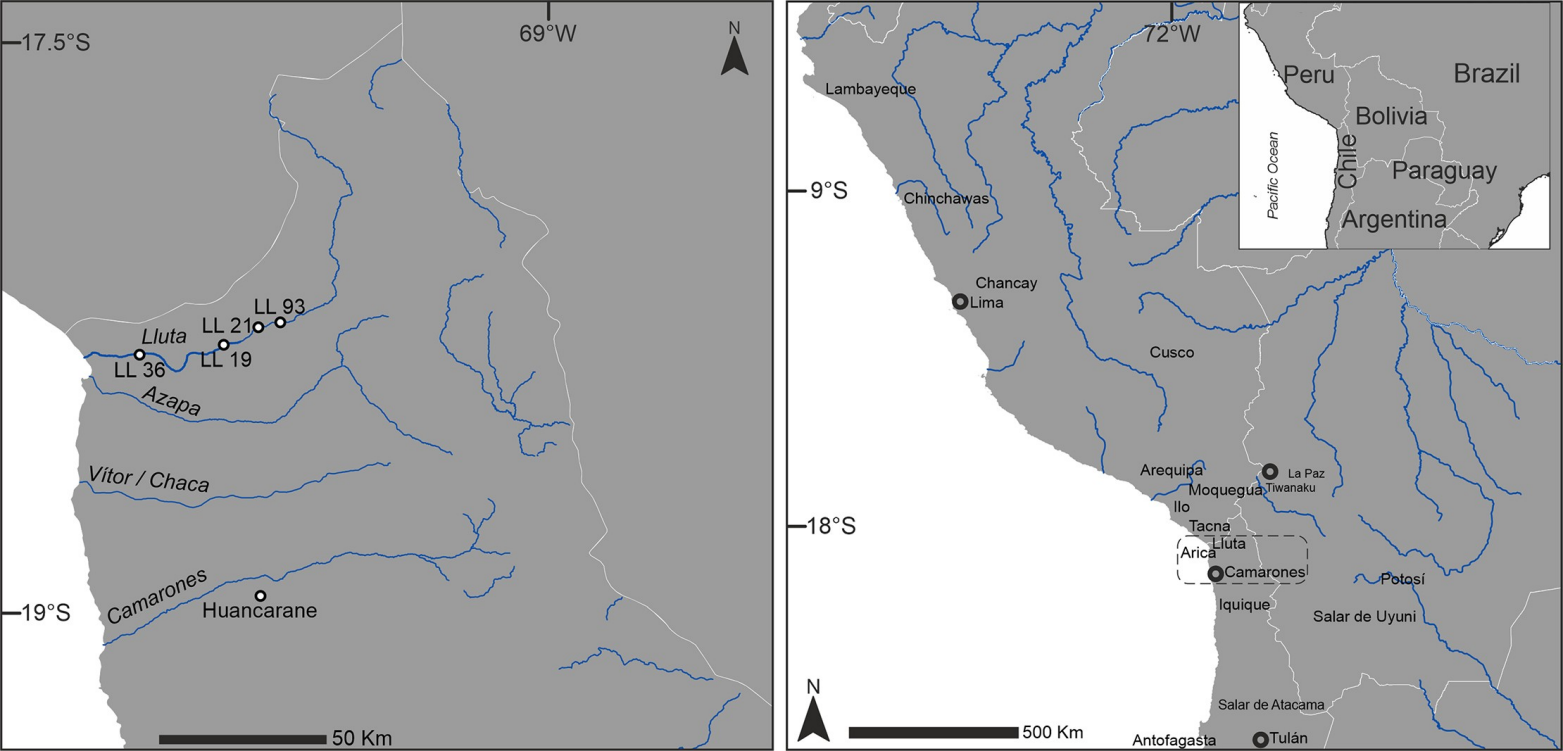
Map of the study area. Relevant toponyms mentioned in the text and study area (box) with archaeological sites including in this study.

The samples analyzed in this study are from five small villages: four from the Lluta Valley (Rosario 2 [LL36], Sora Sur [LL19], Poblado Millune [LL21], and Vinto 1–2 [LL93]), and one from the Camarones Valley (Poblado Huancarane 1) (Fig 1). LL36 was excavated in 1995 by Calogero Santoro [47]. LL19, LL21, and LL93 were excavated in 2011–2012 [48]. Huancarane was excavated in 1978 by Niemeyer and Schiappacasse and its collection was studied in 2015 at the Museo Nacional de Historia Natural (Santiago de Chile). Radiocarbon dates for these sites are summarized in Table 1. All dates listed in Table 1 were calibrated in Calib v7.0.4 [49] using SHCal13 [50].

**Table 1:** AMS radiocarbon dates obtained from the studied sites.

AMS Lab	AMS Lab ID	Material Sample	^14^C Age years BP	±	Site	Precint/ Square	cal AD (1σ Range, SHCAL13)	Probability	Reference
Beta	336650	Plant (seed)	290	30	LL-019	URC1-I	1626–1668	0.814	[48]
Beta	294874	Charcoal	590	30	LL-019	URC-1/III-IV	1393–1424	1	[48]
Beta	336649	Plant	590	30	LL-019	URC-1/III-IV	1393–1424	1	[48]
Beta	294873	Charcoal	640	30	LL-019	URC-1/II	1319–1351	0.714	[48]
Beta	336652	Plant (seed)	390	30	LL-021	URS2-I	1575–1622	0.506	[48]
Beta	180800	Charcoal	450	60	LL-021	R11/N5	1432–1507	0.755	[48]
Beta	294875	Charcoal	450	30	LL-021	URS2-I	1445–1489	1	[48]
Beta	336651	Maize (seed)	490	30	LL-021	URS2-II	1432–1456	1	[48]
Beta	336656	Maize (seed)	360	30	LL-093	URC2-II	1504–1589	0.875	[48]
Beta	294877	Charcoal	370	30	LL-093	URC2-II	1537–1599	0.618	[48]
Beta	336657	Plant	390	30	LL-093	URC2-II	1575–1622	0.506	[48]
Beta	336655	Plant (seed)	620	30	LL-093	URC2-III-IV	1323–1346	0.536	[48]
Beta	336654	Maize (seed)	630	30	LL-093	URC1-III-IV	1321–1349	0.636	[48]
Beta	294876	Charcoal	680	30	LL-093	URC2-III-IV	1300–1324	0.381	[48]
DirectAMS	23334	Maize (seed)	380	45	Huan-001	Precint 74	1541–1625	0.722	unpublished
Beta	434056	Basket	530	30	Huan-001	Precint 132	1418–1443	1	unpublished
DirectAMS	23335	Maize (seed)	700	30	Huan-001	Precint 79	1353–1383	0.567	unpublished
Beta	101496	Charcoal	430	80	LL-36	Precint 58-Level 5	1442–1512	0.541	[51]

The archaeological site LL-36 (Rosario 2) is a hamlet located 16 km from the Pacific coast, composed of *totora* (reed) walled rectangular structures raised on artificial platforms reinforced with frontal stone walls, to level the steep slope of the valley [43]. Based on stratigraphy, radiocarbon dating, and ceramic components, it has been estimated that the earliest occupation was brief, corresponding to the Late Intermediate Period. More intense occupation occurred during the subsequent Late Horizon, as the inhabited surface of the site became larger, and midden deposits are more dense. The site is also distinguished by the occurrence of typical Inca goods (mainly ceramics, such as Inca Polychrome and Saxamar or Inca-Pacaje types, and some fragments of *khipu*) and a large number of exotic goods. Domestic remains show consumption of a wide array of marine resources (e.g., mollusks, fish, seaweed, sea stars), terrestrial fauna (e.g., camelids, guinea pigs), wild plants (e.g., molle, *totora*), crops (e.g., cotton, pumpkin, bean, *ch’uño*, and corn), mineral copper beads, basketry, weaving tools, and abundant textile remains. Systematic analysis of ceramics indicates important changes related to the Inca influence [46, 47, 49].

LL-19 (Sora Sur), located 48 km from the Pacific coast, is a small village of circular precincts made of stones [48]. An occupation from the Late Intermediate Period and another from the Late Horizon were found during excavation. Generally, there are no significant differences between the material culture and biological remains from these two periods, which consist of wild plant remains (e.g., *Schinus molle*, *Prosopis* sp.), crops (*Lagenaria* sp. and *Zea mays*), and lithic artifacts. Camelids and other terrestrial fauna such as canids and rodents (e.g., *Chaetophractus nationi*, *Chinchilla* sp., Cricetidae) are scarce. Marine fauna is also represented by a small number of mollusks (*Choromytilus chorus*, *Littorina peruviana*, Mytílido, *Perumytilus purpuratus*) and unidentified fish. Some textiles (mainly yarns) were also recovered. Local (e.g., Pocoma, San Miguel, and Gentilar) and highland (e.g., Black on Red and Charcollo) pottery types occur in both strata, with Inca Imperial ceramics limited to the Late Horizon stratum.

LL-21 (Poblado Millune, 54 km from the Pacific coast) is a complex village of circular precincts made of stones, organized in differentiated sectors: habitation, storage, and funerary [48]. The excavated deposit contained an occupation that was assigned to the Late Horizon. The materials recovered include local (e.g., San Miguel), highland (e.g., Chilpe, Charcollo), and Inca Imperial pottery, lithic and plant artifacts, and textiles (mainly yarns). There is a predominance of plant remains, with both wild (e.g., *Prosopis* sp., *Schinus molle*, *Equisetum* sp., *Typha angustifolia*) and domesticated (e.g., *Cucurbita* sp., *Gossypium* sp., *Lagenaria* sp., *Zea mays*) species represented. Faunal remains are relatively scarce, with the assemblage consisting of terrestrial mammals (e.g., Camelidae, *Lama* sp., *Lycalopex* sp., Chinchillidae, Cricetidae), mollusks (e.g., *Choromytilus chorus*, *Perumytilus purpuratus*), and finfish (*Trachurus murphyi*).

LL-93 (Vinto 1–2, 57 km from the Pacific coast) is a hamlet composed of residential, funerary, storage, and public use structures (*kancha*, i.e., enclosure) [48]. Three occupations were identified, dating to: the Late Intermediate Period, Late Horizon, and Colonial Hispano-Indigenous Period. The material culture throughout these periods does not show significant changes in composition and consists of lithic artifacts, and mollusk and plant tools. Ecofacts include gathered plant remains (e.g., *Prosopis* sp., *Phragmites australis*, *Schinus molle*, *Tessaria abdinthioides*, *Equisetum* sp., *Scirpus* sp., *Typha angustifolia*, *Tessaria abdinthioides*), crops (*Cucurbita* sp., *Gossypium* sp., *Zea mays*, *Capsicum* sp.), camelids (Camelidae, *Lama* sp., *Vicugna* sp.), rodents (*Cavia* sp., Caviomorpho, Chinchillidae, Cricetidae, *Lagidium* sp.), and other unidentified mammals. Coastal resource procurement is evidenced by the presence of mollusks (*Choromytilus chorus*, *Scurria scurra*, *Tegula* spp). Some textiles (mainly yarns) were also recovered. The ceramics include highland types (e.g. Charcollo) and, in late periods, Inca Imperial styles. The colonial occupation of the site was documented by the presence of skeletal remains of Old World taxa and Hispanic pottery.

Huancarane 1 (60 km from the Pacific coast) is a village composed of stone structures, with differentiated areas (habitation, storages, and funerary) architecturally similar to the Lluta villages described above [48]. The archaeological material comes from the excavation of habitation and storage structures, although stratigraphic information is lacking. The evidence recovered from the excavations includes cultivated (*Zea mays*, *Phaseolus* sp., *Lagenaria* sp., *Gossypium* sp.), and wild plant remains (*Prosopis* sp.), lithic artifacts, wood, and textiles [52]. Some fiber samples were microscopically analyzed and identified as llama, guanaco, and vizcacha (*Lagidium* sp.) by Niemeyer and Schiappacasse [52]. The pottery includes LIP Pica-Tarapacá, Arica, Altiplano and Inca components [53]. Marine mollusks (*Choromytilus chorus*, *Oliva peruviana*, *Cryphiops caementarius*) and freshwater crustaceans (*Cryphiops caementarius*) were recorded, but both were scarce. On the contrary, abundant remains of terrestrial fauna were recovered, including camelids, rodents (*Chinchilla* sp), and other unidentified mammals.

### Isotopic context

The application of isotopic analysis in the study of animal husbandry and the trade in animal products is a rapidly growing area of inquiry. Some isotope systems, such as hydrogen, oxygen, strontium, and lead, record the location in which an animal lived [54], and these techniques have been applied with some regularity in archaeological contexts to address the trade in animal products [55–59]. Stable carbon and nitrogen isotope compositions are not tied to geography in the same way as these other isotope systems, but in certain regions where environmental variation is large over relatively small spatial scales or particular feeding practices that are isotopically unique exist, carbon and nitrogen isotopes can be an effective means of assessing the locality of animals and animal products in the archaeological record [31, 32, 60].

The carbon and nitrogen isotope compositions of a consumer’s tissues reflect the average carbon and nitrogen isotope compositions of the foods consumed during the period of tissue formation [61, 62]. The carbon in bone collagen is predominantly routed from dietary protein [63, 64]. Herbivore tissue *δ*^13^C and *δ*^15^N therefore reflect the *δ*^13^C and *δ*^15^N of the plants that they consumed [65, 66], which are in turn sensitive to a number of environmental parameters [67, 68].

Photosynthetic pathway is the primary mechanism influencing the *δ*^13^C values of plants. Excluding plants growing under dense forest canopy, C_3_ plants have *δ*^13^C values that range between −35 and −20 ‰ with a mean of c. −27 ‰; C_4_ plants (predominantly tropical grasses) have *δ*^13^C values that range between −15 and −7 ‰ with a mean of −12 ‰; CAM plants (cacti, succulents, and epiphytes) have *δ*^13^C values that range between −22 and −10 ‰ [69–71]. C_3_ plant *δ*^13^C values are sensitive to environmental variation through influences on the ratio of ambient to intercellular partial pressure of CO_2_ [67, 72]. The *δ*^13^C values of C_3_ plants are correlated with water availability such that plants growing in arid conditions have higher *δ*^13^C values than those growing in wetter conditions [73–76]. Water availability does not appear to influence C_4_ plant *δ*^13^C [75, 77], which are generally less sensitive to environmental variation and exhibit a much narrower range of *δ*^13^C values than C_3_ plants [72]. The entire region considered for this study is characterized by low water availability, therefore, wild C_3_ plants will have *δ*^13^C values that are higher than the global average. In a series of altitudinal transects between 22°S and 25°S in northern Chile, Quade et al. [78] found the average *δ*^13^C of C_3_ plants to be −23.1 ‰ (c. −21.6 ‰ after accounting for the Suess Effect). Similarly, Rundel et al. [79] found the average *δ*^13^C of C_3_ plants growing in the pre-puna shrubland zone (c. 3,550 masl) at 18°S to be −24.1 ‰ (c. −22.8 ‰ after accounting for the Suess Effect). The *δ*^13^C of the overall biomass at high altitudes (>3,500 masl) is still lower than at low altitudes because of the rarity of C_4_ plants [80, 81]. Despite the rarity of C_4_ plants in the arid highland regions of northern Chile, the *δ*^13^C values of herbivores consuming pure C_3_-diets should be relatively high. In In this region, herbivore *δ*^13^C values of −18 ‰ do not necessarily imply the consumption of any C_4_ plants assuming a consumer-diet trophic enrichment factor (*Δ*^13^C) of c. +5 ‰ [82]. Soil salinity also influences the *δ*^13^C values of plants, with high values occurring with increasing soil salinity [83–85]. Finally, plants growing under dense forest canopies have lower *δ*^13^C values than plants growing in open habitats [86–88], although this particular variable is not relevant for this study as the area lacks the necessary tree cover.

Plant tissue *δ*^15^N values are determined primarily by the N source [68], the most important of which are mineralized N (NO_3_^−^ and NH_4_^+^) and atmospheric N_2_ [89, 90]. Some plants in environments with low mineralization rates (typically boreal and arctic environments) also rely to a significant extent on organic N [91]. There is essentially no discrimination against ^15^N during the conversion of N_2_ to NH_3_, a process that is known as biological nitrogen fixation (BNF [92, 93]). Plants that have the capacity to rely on N_2_ through symbiotic associations with bacteria (primarily legumes, Fabaceae) therefore tend to have *δ*^15^N close to that of atmospheric N_2_ [94–96], which is 0 ‰ [97]. The reliance on N provided through these symbiotic associations is, however, metabolically expensive and if soil N availability is high, legumes and other taxa capable of BNF will rely on mineralized N sources [94].

The types of mycorrhizal associations that plants form are also an important factor in influencing plant *δ*^15^N, with plant values being highest in non-mycorrhizal and arbuscular mycorrhizal (AM) plants, lower in ectomycorrhizal (EcM) plants, and lowest in ericoid (ErM) myccorhizal plants [98, 99]. Generally there is latitudinal variation in the distribution of mycorrhizal types, following trends in temperature and soil N mineralization rates, with AM plants dominating in temperate grasslands and savannahs, EcM plants dominating in temperate forests and boreal taiga, and ErM dominating in high latitude tundra [100, 101]. Altitudinal trends in mycorrhizal associations are less well studied, but it appears that AM abundance declines with altitude, EcM abundance peaks at mid altitudes (1,000 to 2,500 masl), and the abundance of ErM is generally limited by the distribution of their plant partners, the Ericaceae, which tend to occur in acidic soils with low N availability [102].

In agricultural systems, additional N may be added to the soil through fertilization. The use of N derived from fertilizers has the capacity to increase plant *δ*^15^N values by a few ‰ for manure derived from domestic herbivores such as cattle or camelids [103–105] to greater than 20 ‰ for seabird guano [94, 105, 106]. Both types of fertilizer would have been potentially available in the study area and in fact the only convincing evidence of the prehistoric use of seabird guano as a fertilizer to date comes from the Atacama Desert immediately south of our study area [107].

Plant *δ*^15^N tends to be positively correlated with temperature [108, 109] and negatively correlated with water availability [65, 74, 75, 98, 108, 110]. Because average temperature decreases and annual precipitation increases moving from the Pacific coast and into the highlands of Peru and Chile, plants growing at high altitudes have lower *δ*^15^N than those growing at lower altitudes [111]. That said, the high altitude *puna* of northern Chile is distinct from that which exists in Peru in that it is far more arid. This region does receive more precipitation than the coast, but the region is still arid, with the northern Chilean dry *puna* receiving 300−350 mm of annual precipitation [112]. Despite the aridity of the *puna*, studies conducted in the hyperarid salt *puna* around 23.5°S [113] and in the Argentine dry *puna* [114] found plant *δ*^15^N values to be comparable to those observed in the highlands of northern Peru (c. +2 to +6 ‰) [111]. Given that herbivores tissue *δ*^15^N values are 3 to 4 ‰ higher than the plants that they consume [115], we would expect camelids that lived in the *puna* to have tissue *δ*^15^N values between +5 and +9 ‰. This expectation requires testing through the collection of isotopic data from archaeological camelids recovered from sites in the highlands of northern Chile. For animals living in the low altitude coastal river valleys of northern Chile we expect much higher *δ*^13^C and *δ*^15^N values than those living in the highlands driven primarily by the influence of aridity on plant *δ*^15^N values and the greater abundance of C_4_ plants, particularly cultivated maize, which could have been used as fodder for camelids.

## Materials and methods

### Sample preparation

Bone collagen was extracted by demineralizing chunks of bone (c. 200 mg) in 0.5 M HCl at room temperature under constant motion (orbital shaker) for 7 days with periodic changing of the acid solution. After demineralization, the samples were rinsed to neutrality with Type I water and then those samples that were darkly colored were treated with 0.1 M NaOH for 20 min under constant motion (orbital shaker). If after 20 min the solution changed color, the solution was removed and fresh NaOH solution was added. Once there was no color change in the solution after 20 min, the samples were rinsed to neutrality with Type I water. The samples were then heated at 75°C for 36 h in 4 ml of 10^−3^ M HCl. After heating the solution containing the water-soluble collagen was transferred to a vial and freeze-dried.

The textile samples were cleaned of any visible particulate matter with a dental pick. The samples were then sonicated in Type I water for 60 min, centrifuged and air-dried. The textiles samples were then sonicated in 2:1 chloroform-methanol (*v*/*v*) for 60 min, centrifuged and air-dried.

### Isotope ratio mass spectrometry

Carbon and nitrogen isotopic and elemental compositions were determined using a Nu Horizon continuous flow isotope ratio mass spectrometer couple to a EuroEA 3000 elemental analyzer at Trent University. Sample isotopic compositions were calibrated relative to VPDB (*δ*^13^C) and AIR (*δ*^15^N) using USGS40 and USGS41a or USGS66 [116–118] (S1 Appendix). Elemental compositions were calibrated using USGS40. Analytical uncertainty was assessed using four internal standards interspersed among the samples and 20% of the samples were analyzed in duplicate (S1 Appendix). Standard uncertainty was determined to be ±0.20 ‰ for *δ*^13^C and ±0.29 ‰ for *δ*^15^N [119].

### Sample integrity

**Fig 2:**
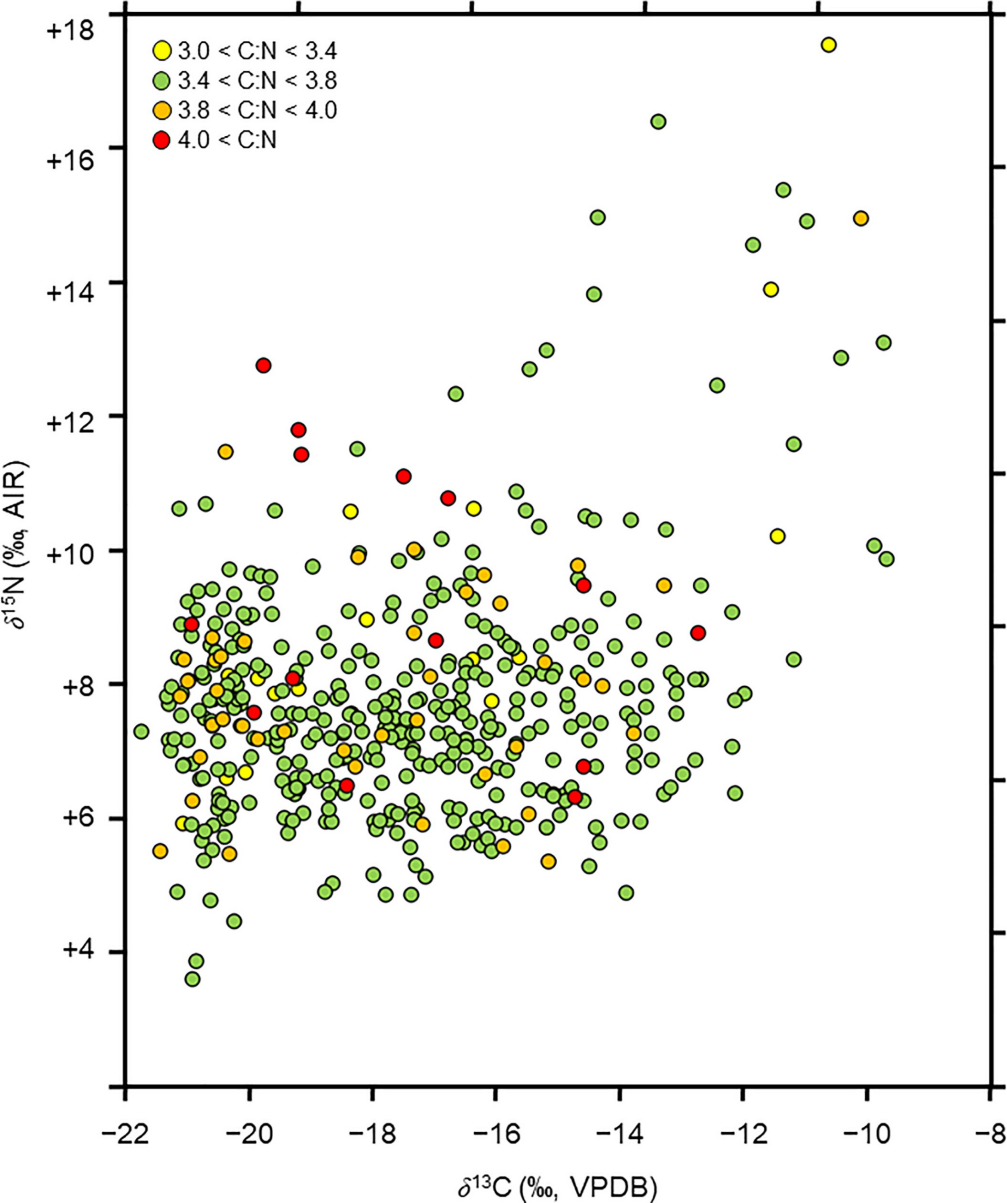
Relationship between isotopic and elemental composition of camelid fiber textiles. Carbon and nitrogen isotopic compositions of textiles analyzed in this study, as well as other camelid fiber samples from previously published papers [26, 31, 32]. Samples are colored according to their atomic C:N ratio.

Quality criteria are not as well established for ancient keratin as they are for collagen. Boudin et al. [120] found an atomic C:N ratio range of 3.4−3.8 to be reliable within the context of ^14^C dating of wool. von Holstein et al. [121] found minimal changes to the carbon and nitrogen isotope compositions of wool textiles undergoing experimental degradation. Our approach to monitor for textile samples with unreliable isotopic compositions altered by post-depositional processes was informed by the approaches of Ambrose [122] and DeNiro [123] for bone collagen. DeNiro defined an acceptable range of 2.9–3.6 for unaltered collagen by examining the elemental and isotopic compositions for different taxa and noting that the collagen samples with C:N ratios outside the range of 2.9–3.6 tended to produce isotopic compositions that were too high or too low (S1 Appendix). Subsequently, others have discussed other quality control measures [122, 124–126], but DeNiro’s range of 2.9–3.6 for atomic C:N ratios remains the most frequently cited quality control measure in isotopic studies of ancient collagen [119]. We have taken a similar approach to DeNiro and compared the isotopic compositions and the atomic C:N ratios of the textiles analyzed in this study, as well as several others from the Andean region (Fig 2).

The data produced by DeNiro for bone collagen show clear differences in isotopic compositions between samples with C:N ratios within vs. outside of the 2.9–3.6 range (S1 Appendix). Specifically, the *δ*^13^C values tend to be especially low when the C:N ratios are outside of the 2.9–3.6 range and the *δ*^15^N values are much more variable (may be too high or too low). For archaeological camelid fiber, the samples with relatively high and low C:N ratios were not distinct, unlike those presented for DeNiro’s bone collagen data. This difference may be because highly degraded keratin does not survive in the burial environment while bones containing virtually no residual organic matter are reasonably common.

Although Boudin et al. [120] defined an atomic C:N range of 3.4–3.8 as being acceptable for radiocarbon dating sheep’s wool, modern camelid fiber samples produced a range of atomic C:N ratios between 3.10 and 3.45 (*n* = 85) [24]. It therefore seems unreasonable to exclude data based on criteria defined for another taxon that produces a distinct type of fiber [127]. For the dataset presented in Fig 2, encompassing 452 analyzed camelid fiber samples from Peru and Chile, a significant number of samples with atomic C:N ratios greater than 4 produced relatively low *δ*^13^C and high *δ*^15^N values. Fourteen samples produced *δ*^13^C values less than −16 ‰ and *δ*^15^N values greater than +10 ‰. Of these fourteen, five (36%) had C:N ratios that were greater than 4, while the number of fiber samples in the entire dataset producing C:N ratios greater than 4 was 14 (3% of the total sample). On this basis, we excluded any samples with an atomic C:N ratio over 4 from our analysis.

### Data treatment

When comparisons were made between bone collagen and textile isotopic compositions, the textile *δ*^13^C values were adjusted by +1.3 ‰ to account for inter-tissue differences in diet-tissue fractionation. The isotopic composition of bone collagen represents the weighted average of foods consumed over a period of several years [128]. While hair grows incrementally and is inert once formed, the nature of textile samples is such that the period of time represented by an isotopic measurement cannot be determined, nor is it certain that a single animal is represented in any particular fiber sample. Therefore, the isotopic compositions of textile samples represent an average dietary intake over an indeterminate amount of time. It is therefore important to generate larger numbers of isotopic measurements of textiles than of bone collagen to adequately capture the variability within a single site or context.

To assess differences in the isotopic compositions between periods or regions, one of the following tests were used: Student’s t-test (normally distributed, equal variances), Welch’s t-test (normally distributed, unequal variances), or Mann Whitney U test (non-normally distributed). Normality was assessed using a Shapiro-Wilk test. Equality of variance was assessed using Levene’s test. The presence of discrete isotopic groups in the textile dataset was assessed using an unweighted pair group method with arithmetic mean cluster analysis with a Euclidean distance function.

The amount of bivariate isotopic variation was estimated using the standard bivariate ellipse in the SIBER package [129]. Ellipse sizes reported in the text are standard ellipse areas corrected for sample size (SEA_*c*_). Comparisons between ellipses were performed with 10^4^ iterative draws (SEA_*b*_) with the results being expressed as the percentage of computed ellipses for Group 1 that are larger than the computed ellipses for Group 2; a value of 0.50 indicates the two ellipses are identical in size, while a value of 1 or 0 would indicate that the ellipses for Group 1 are always larger or smaller than those for Group 2.

The cluster analysis was performed using Past 3.20. The SIBER calculations were performed in R 3.0.3; the scripts are included in the supplementary information (S1 File). All other statistical tests were performed using IBM SPSS 23.

## Results

### Bone collagen

**Fig 3:**
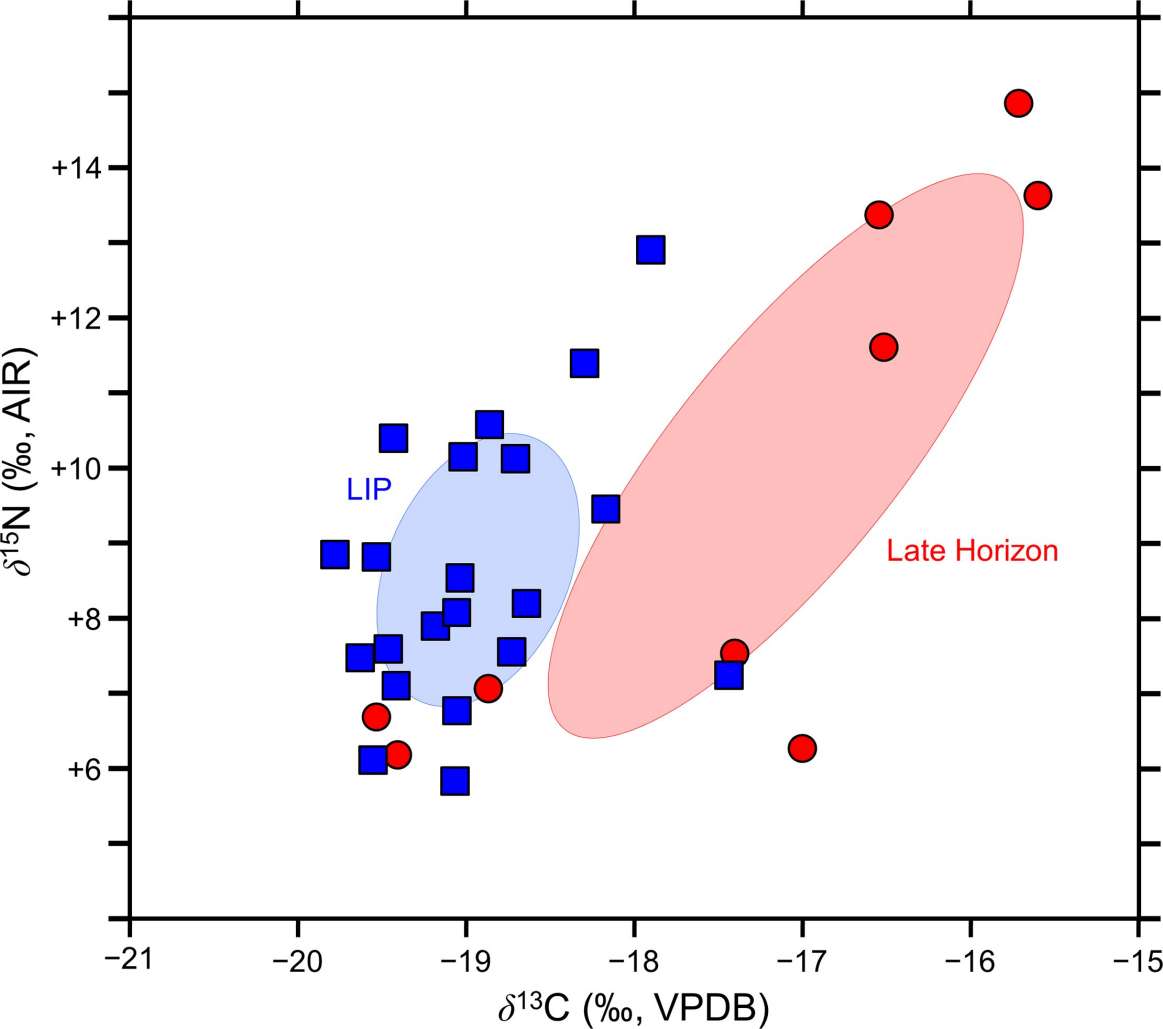
Bone collagen *δ*^13^C and *δ*^15^N values for camelids from the Lluta Valley along with the standard bivariate ellipses for the two time periods. Late Intermediate Period samples are indicated by squares and Late Horizon samples are indicated by circles.

The bone collagen *δ*^13^C and *δ*^15^N values are presented in S1 Table and Fig 3. The camelids from the Late Horizon possessed significantly higher *δ*^13^C values than those dating to the LIP (Welch’s t-test; *t* = −3.46, *df* = 8.59, *p* = 0.008); the *δ*^15^N values did not differ between the two periods (Welch’s t-test; *t* = −0.46, *df* = 9.36, *p* = 0.65). There was no overlap between the standard ellipses generated for the camelid bone collagen dating to the LIP and LH (Fig 3). The bivariate isotopic variation observed for the LH camelids was much greater than for the LIP camelids (SEA_c_ of 10.4 compared to 3.0), with the modeled ellipses of the LH group being larger than the LIP group in 99% of the comparisons.

### Textiles

**Fig 4:**
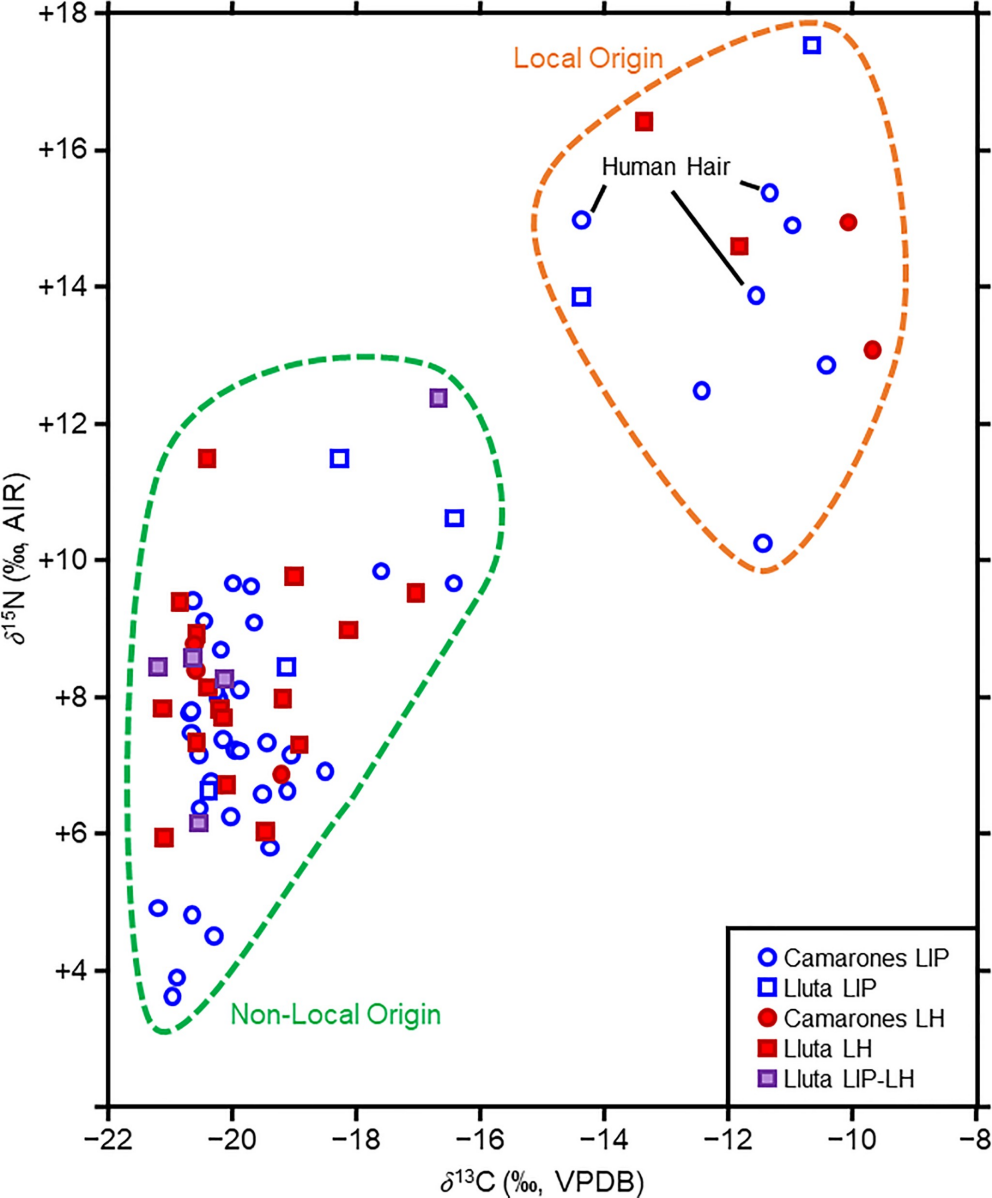
Stable carbon and nitrogen isotope compositions of all of the textile samples analyzed.

**Fig 5:**
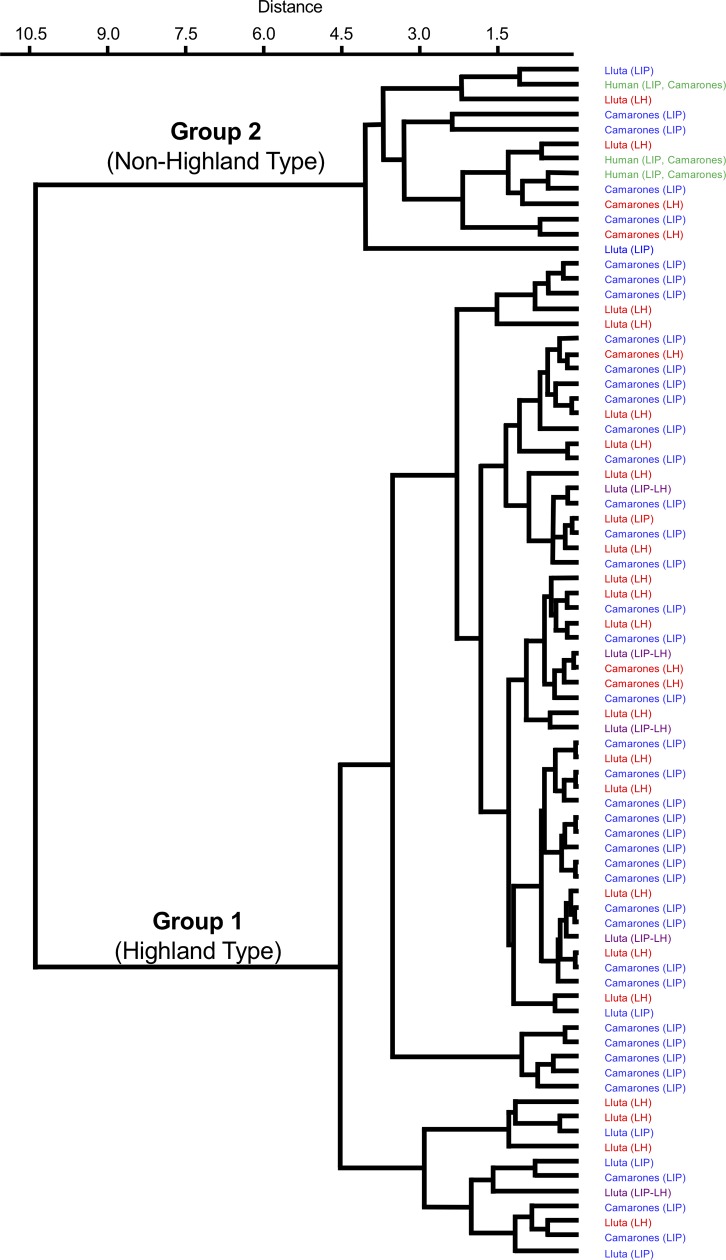
Results of the cluster analysis for the textile and human hair samples.

The textile carbon and nitrogen isotopic and elemental compositions are presented in S2 Table. There were two distinct groups of textiles, one with lower *δ*^13^C and *δ*^15^N values and one with higher *δ*^13^C and *δ*^15^N values (Fig 4); these groupings were confirmed with a cluster analysis (Fig 5). For the purposes of further analysis, each cluster was considered a distinct data set and they are referred to as Textile Group 1 (*δ*^13^C = −19.77±1.05 ‰, *δ*^15^N = 8.19±2.07 ‰, *n* = 60) and Textile Group 2 (*δ*^13^C = −14.44±1.55 ‰, *δ*^15^N = +13.97±1.63 ‰, *n* = 12). Relative to the overall textile sample, the two groups contained similar proportions of samples from the Lluta (45% of Group 1, 44% of overall sample) and Camarones (55% of Group 1, 56% of overall sample) valleys, as well as the LIP (58% of Group 1, 57% of overall sample) and LH (34% of Group 1, 36% of overall sample). Therefore, the presence of these two textile groups could not be explained by a regional or temporal effect.

Within Textile Group 1, samples from the LIP and LH did not differ significantly with respect to their *δ*^13^C (Mann Whitney U test; *U* = 385.5, *p* = 0.36) or *δ*^15^N (Student’s t-test; *t* = 1.70, *df* = 61, *p* = 0.09). The textile samples within Group 1 from the Lluta Valley had significantly higher *δ*^15^N values than those from the Camarones Valley (Student’s t-test; *t* = 3.11, *df* = 66, *p* = 0.003), although there were no significant differences in *δ*^13^C between the two valleys (Mann Whitney U test; *U* = 541, *p* = 0.86).

**Fig 6:**
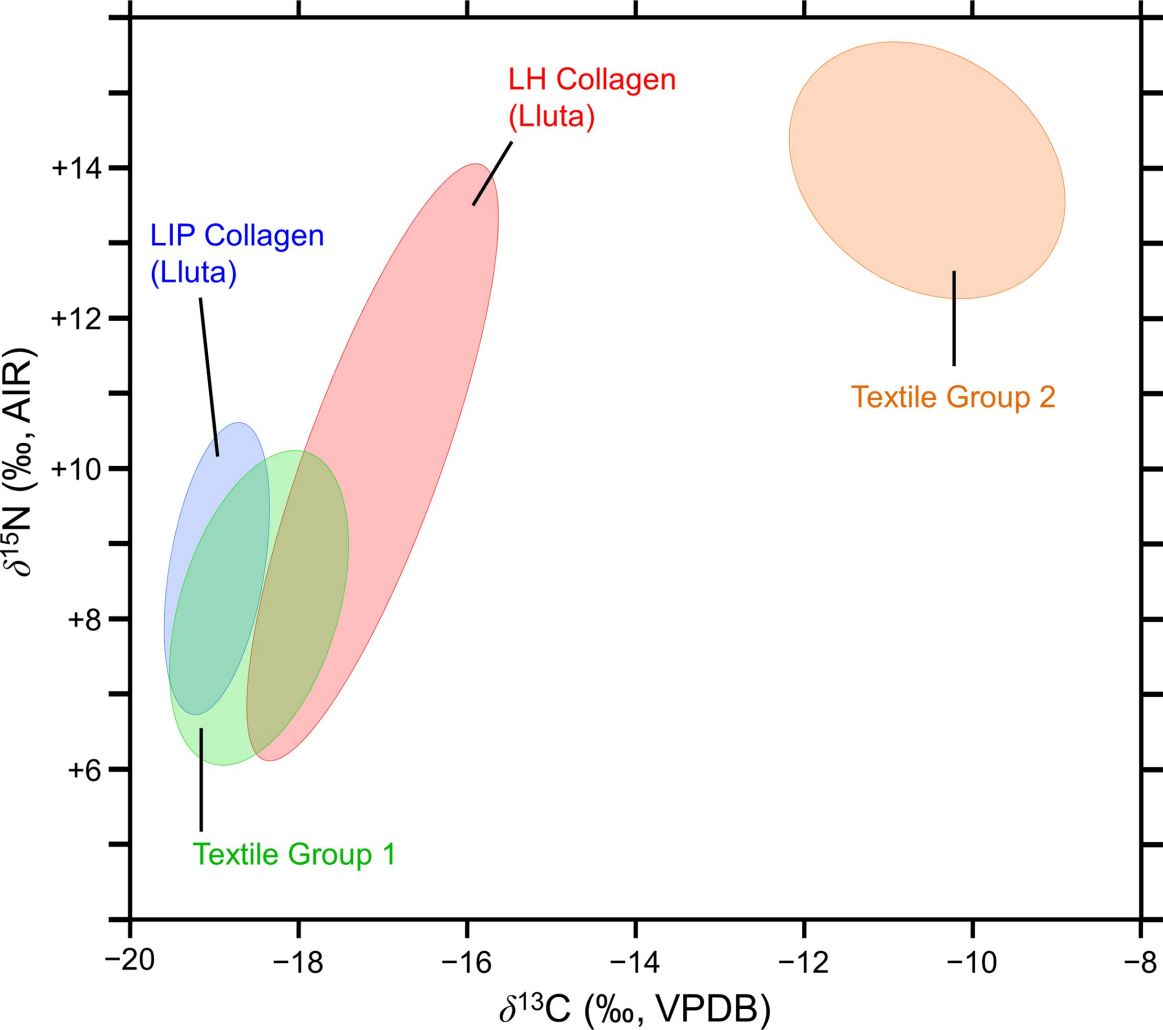
Comparison of the camelid bone collagen and textile isotopic compositions. Comparisons are based on standard ellipse areas for the camelid bone collagen from the LIP and LH in the Lluta Valley and the two textile groups (as determined by the cluster analysis) for the Lluta and Camarones Valleys. The textile *δ*^13^C values have been adjusted by +1.3 ‰ to make them directly comparable with the bone collagen.

After adjusting for differences in fractionation between tissues, Textile Group 1 had similar isotopic compositions to the camelid bone collagen samples from the sites in the Lluta Valley, falling between the LIP and LH bone collagen ellipses (Fig 6). Textile Group 2 possessed markedly higher *δ*^13^C and *δ*^15^N values than either the LIP or LH bone collagen datasets from the Lluta Valley (Fig 6).

Unspun fleece samples had higher *δ*^13^C and *δ*^15^N values (*δ*^13^C = −16.57±4.52 ‰, *δ*^15^N = +9.33±3.70 ‰, *n* = 15) than spun yarns (*δ*^13^C = −19.19±2.40 ‰, *δ*^15^N = +8.54±2.56 ‰, *n* = 56). This difference was statistically significant for *δ*^13^C (*U* = 236, *p* = 0.01) but not for *δ*^15^N (*U* = 409, *p* = 0.88). A higher proportion of the Group 2 textiles consisted of unspun yarns (*n* = 5 or 50%) relative to Group 1 (*n* = 10 or 14%).

## Discussion

### Camelids originating in the highlands

**Fig 7:**
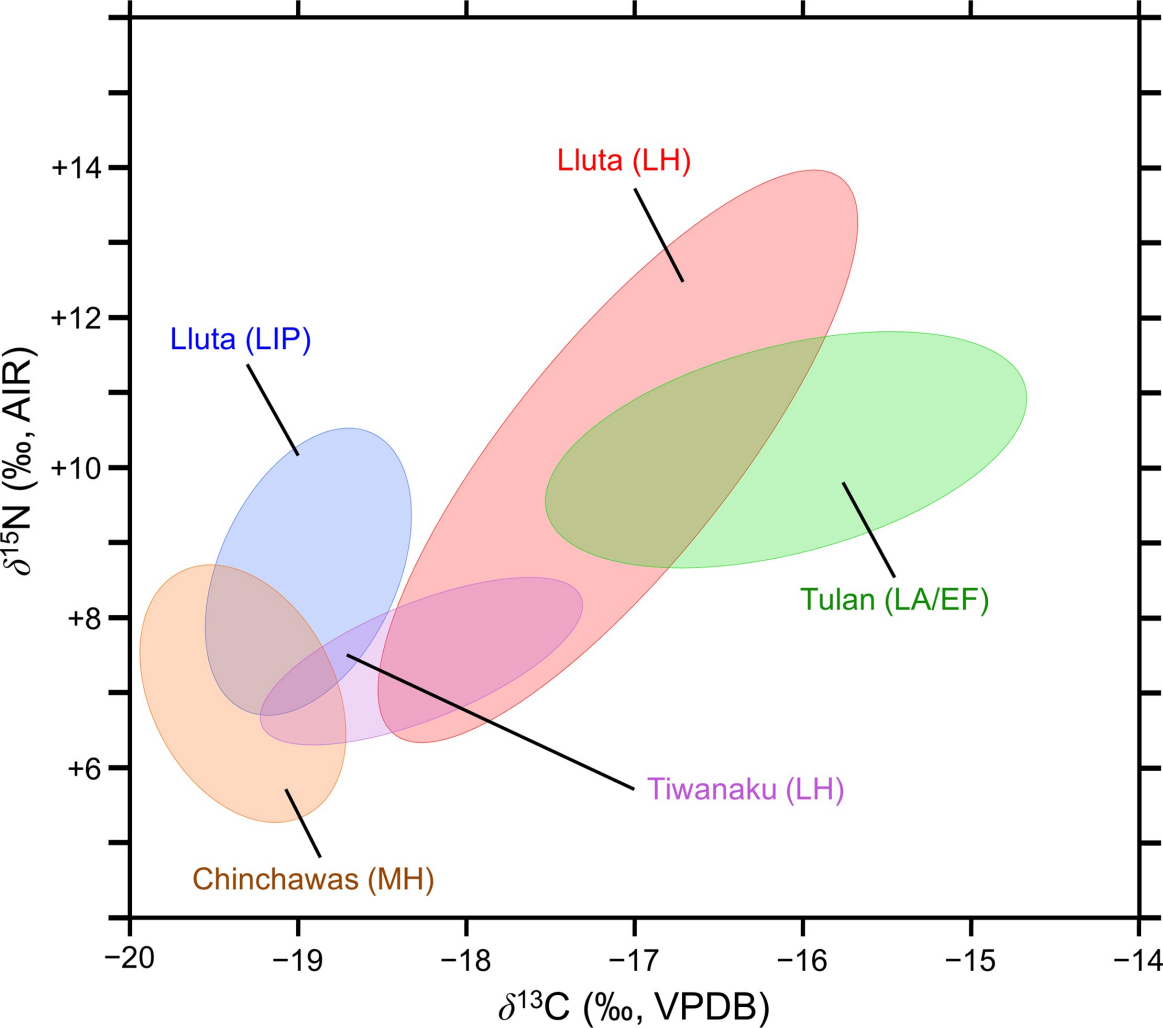
Interregional comparison of camelid bone collagen isotopic compositions. Standard bivariate ellipses for the camelid bone collagen from the Lluta Valley and various sites where the camelids are believed to have lived in the highlands: Chinchawas [31], Tiwanaku [131], and Tulan (Late Archaic/Early Formative) [132].

The bone collagen *δ*^13^C and *δ*^15^N values of the Lluta Valley camelids are consistent with a highland origin. The LIP and LH camelid bone collagen *δ*^13^C and *δ*^15^N values are summarized as standard bivariate ellipses and compared to camelids from other relevant sites where the camelids are known to have been raised in the highlands (Fig 7). Among the three comparative sites, there is a pattern of increasing *δ*^13^C values with increasing latitude, with the lowest values at Chinchawas (~9.5°S), intermediate values at Tiwanaku (~16.5°S), and the highest values for sites on the Tulan transect (~23.5°S). This pattern likely has nothing to do with increasing quantities of C_4_ plants in the diet with increasing latitude, but with increasingly high *δ*^13^C values as the *puna* environment becomes drier moving north to south, transitioning from moist *puna* to dry *puna* to salt *puna* [112, 130]. In light of their *δ*^15^N values, both the LIP and LH camelids from the Lluta Valley sites lived in areas that were more arid than those at either Tiwanaku or Chinchawas. The northern Chilean *puna* is the most likely origin for these camelids, but the stark isotopic difference between the LIP and LH camelids from the Lluta Valley sites requires further discussion.

### Changes in geographic origins of camelids during the late horizon

The LH camelids from the Lluta Valley can be divided into two groups, one with lower *δ*^13^C and *δ*^15^N values, and one with higher *δ*^13^C and *δ*^15^N values; the latter group is largely responsible for the difference between the two periods (Fig 3). This apparent division among the LH group may be driven by these camelids originating in two distinct geographic regions, one of which is much drier (for the camelids with the higher *δ*^13^C and *δ*^15^N values) than the other (for the camelids with the lower *δ*^13^C and *δ*^15^N values). Alternatively, camelids may have been drawn from a more diverse range of environments overall during the LH, but because of the small size of our sample, we have simply failed to capture the full range of this variability. Regardless, our data suggest that there was a shift in the range of environments from which camelids were obtained during the LH relative to the LIP.

During the LIP, we suggest that the inhabitants of the Lluta Valley acquired their camelid meat from the *puna* located adjacent to the northern valleys. In the northern valleys, the LIP was a time characterized by regionalization in the wake of the Tiwanaku polity. One crucial point of debate has been whether or not there was physical resettlement of groups from the *altiplano* into the northern valleys following Tiwanaku’s demise [133, 134]. In a recent synthesis, Muñoz et al. [42] conclude that, although there were Tiwanaku influences during this period, there is no archaeological or bioanthropological evidence to support the presence of Tiwanaku colonies in northernmost Chile. The influence of Tiwanaku is mainly expressed in Cabuza and Tiwanaku pottery types. Cabuza was a locally produced and consumed style that attempted to emulate Tiwanaku forms and decoration. The Tiwanaku style reproduces forms and decorations of the expansive Tiwanaku Phase V, but has strong influences from Moquegua, suggesting its provenience in these valley in southern Peru, rather than the *altiplano* [135, 136]. Recently, radiocarbon dating carried out in Cabuza funerary contexts indicates that most of the dates fall within the LIP, suggesting that the Tiwanaku influence may have been associated with people deserting the Titicaca Basin or colonies in Moquegua following Tiwanaku’s demise [137]. Despite the lack of a strong influence of Tiwanaku on the northern Chilean valleys, the isotopic data from the LIP camelids suggests connections with the *altiplano*. During the LIP, a variety of *altiplanic* goods (e.g., feathers, copper, obsidian, sulfur, pottery) have been systematically recorded in lowland settlements possibly as a consequence of social interaction networks, which were intensified during this period [41, 138]. Camelids could certainly have been an important part of these interregional networks of interaction and exchange, particularly in light of the fact that the sites analyzed in this study are 40 to 65 km from the contiguous *altiplano*.

The fact that the LIP and LH camelids from the Lluta Valley were characterized by distinct isotopic compositions suggests either a change in the geographical origin of the camelids or a change in the way in which they were managed. These could be a consequence of the Inca presence in the lowland valleys of northern Chile. Although no investment in state infrastructure is present, significant changes in the ways of life of the local communities, reorganization of the economy and alterations in the political systems have been recorded as result of the Inca control over this area. These changes are expressed in the incorporation of conspicuous Inca prestige goods (e.g., *aribalos*, *khipu*, *unku*, *tumi*), the concentration of populations in larger villages, alterations of health conditions (intestinal parasites) as a result of overcrowded residences, and the intensification of maize and textile production, which demonstrate clear alterations to the local way of life, although without a visible impact on the architecture [46, 139].

During the LH, rights over resources and lands were claimed as state property by Tawantinsuyu (the Inca Empire) [140]. Camelids were strictly controlled and regulated by the Inca, with three different types of herds: state, church, and community [12]. There were also exclusive regulations for the use of hunting grounds of wild camelids, especially vicuña [141]. Additionally, the Inca state employed redistribution as a socio-political and ideological mechanism to control local populations, although a diverse range of economic structures almost certainly existed within Tawantinsuyu [142]. Among the goods distributed to local communities, wild and domesticated camelids are frequently mentioned in the chronicles [143]. Murra [144] specifies that the camelids redistributed by the state did not come from the community herds, but from the state herds. Chroniclers agree that relatively little camelid meat was consumed during Inca times, except at feasts and ceremonies, although this assertion requires explicit testing with archaeological data [145]. In some Andean regions an increase in the production and/or consumption of camelids during the LH has been identified, typically interpreted within a framework of access to meat and livestock regulated and centralized by the Inca state, sometimes in the form of state-sponsored feasts [140, 145–150].

Consequently, if the Inca state controlled the flow of camelids or their meat among the conquered provinces through state redistribution networks, then the camelids consumed in the Lluta valley may have had a different geographical origin relative to those of the LIP because they were the result of new animal distribution circuits associated with the redistribution networks controlled by the Inca state. During the Inca occupation of Tiwanaku (Pumapunku complex), a pastoralist area *par excellence*, large quantities of camelids were consumed at state-sponsored feasts. Of the basis of isotopic analyses, most of these camelids had a local origin but some of them originated outside of the *altiplano* in the Titicaca Basin [151], possibly because of the diverse origins of camelids from state herds. Although it cannot be established if the camelid bone samples included in this study were associated with state-sponsored commensal meals, some of them could have arrived in the Lluta Valley via redistributive networks.

### Origins of camelid fiber

The fact that the isotopic patterns observed for camelid skeletal remains and fiber from textiles are distinct from one another (Fig 6) suggests that animals raised for different purposes (i.e., meat vs. fiber) originated in different geographic areas, consumed different foods, or a combination of these. During the Late Horizon, camelid herds were segregated according to their function: fiber, meat, and cargo [146, 149]. This is consistent with our results, but that fact that there is no difference in the fiber isotopic compositions between the LIP and LH suggests that the Inca did not influence the geographic regions from which fiber was being obtained in the Lluta and Camarones Valleys. Alternatively, there may have been significant changes in the regions of fiber production and the movement of these goods but if these changes occurred within an isotopically homogenous highland environment, it would not be detectable with the methods applied in this study. For example, if fiber production intensified during the Late Horizon (more fiber produced from the same area of land being grazed [152]), possibly driven by the extraction of tribute from some communities and the development of new categories of specialized weavers [11, 44], this scenario may not have left traces in the isotopic compositions of the textiles. On the other hand, if fiber production extensified (more fiber produced from camelids being raised in a larger number of areas), we might expect to see isotopic evidence of localized camelid husbandry, or at least non-altiplanic camelid husbandry, exclusively in the Late Horizon. Some chronicles indicate that while for local textile needs fiber from the community was used, for the *mit’a* obligations, in contrast, the Inca state provided the fiber, which came from state deposits [143, 144], likely located in the highlands. The presence of weaving tools in households in the valleys of northern Chile suggest that textiles were being manufactured locally even if the fiber was being imported from the highlands.

Considering the separation of herds for fiber and herds for meat as well as the diverse fiber used for local needs versus for *mit’a*, the scenario outlined in the preceding paragraph could suggest different circuits of distribution for meat and fiber during the Late Horizon. Textile production for local consumption, which constitutes the majority of textile samples recovered from these archaeological sites, continued to be dependent on the highlands for fiber, as was during case during the LIP. On the contrary, for the textiles manufactured through *mit’a* labor, we expect that the raw material would have originated in state fiber deposits. Moreover, if the *mit’a* used either state or community fiber, it would likely not be possible to recognize this distinction in the archaeological record because these textiles should have been redirected from local households for state purposes and would not have remained in the area where the fiber was produced [11]. Therefore, *mit’a* textiles should have low archaeological visibility in domestic contexts such as those sampled in this study. In some domestic contexts of the Lluta Valley where important transformations of the Inca state have been verified, including the relocation of populations and the presence of several *khipu*, a remarkable increase in spinning and weaving tools has been identified, which is interpreted as a possible result of textile *mit’a* obligations [44]. Isotopic analyses from contexts such as this may shed light on any changes in fiber production during the Late Horizon.

### Fiber originating outside of the highlands

A subset of the textile samples from both the LIP and LH have isotopic compositions that are inconsistent with camelids that lived in the highlands (Figs 4 and 5). Given the greater importance of llamas for meat and their adaptability to a wider range of environments than alpacas [153], which were primarily fiber producers [20], it is surprising that the textiles, rather than camelid bone samples, appear to have non-highland origins. The issue of raising camelids in the lowlands has not been studied systematically in northern Chile, either through the application of isotopic analysis or traditional archaeological methodologies. Archaeological evidence for the maintenance of camelid herds in the lowlands (although not their permanent presence) includes corral-type structures found in villages [45, 52, 154–156] and concentrations of camelid dung [45, 52, 157–160]. For northern Chile, sixteenth century ethnohistorical accounts describe the maintenance of camelid herds in the lowlands and the tribute of livestock and manufactured textiles to the Spanish Crown by local indigenous groups from Ilo, Tacna, Arica, and Tarapacá [143, 161, 162]. If there were local herds oriented towards fiber production, we would expect to see a significant quantity of unspun fleece with local isotopic signatures. Consistent with this expectations, only 14% of the camelid fibers analyzed had isotopic compositions consistent with a local origin but a disproportionately high number of these (50%) were unspun fleece rather than spun yarns.

In addition to the textiles manufactured from camelid fiber, three textile fragments from Huancarane were made of human hair. These three samples possess *δ*^13^C and *δ*^15^N values that are very similar to the group identified as non-highland camelids (Fig 4). These human hair isotopic compositions are comparable to other LIP and LH data from the region, although humans from northern Chile possess highly variable tissue isotopic compositions [163–167]. The human *δ*^13^C and *δ*^15^N values are consistent with a substantial amount of maize in the diet rather than marine foods as marine organisms from northern Chile possess extremely high *δ*^15^N values due to strong upwelling and denitrification in this region [168, 169]. Human populations in this region that relied on marine foods to a significant degree tend to the highest tissue *δ*^15^N values among any human group, frequently in excess of +20 ‰ or even +25 ‰ [164, 170], substantially higher than those observed in this study: *δ*^15^N = +13.9 ‰, +15.0 ‰, and +15.4 ‰.

The humans from which the hair was obtained were almost certainly locals and the fact that the non-highland textile groups possesses very similar isotopic compositions suggests that these animals may have consumed a similar range of agricultural plants to the humans living in the Camarones Valley. If camelids were kept in these valleys, they would have needed to consume significant quantities of agricultural plants because there is scarce wild vegetation on which they could feed, although there may have been some fields composed of halophytic grasses (*Distichlis spicata*) growing close to the coast at the river mouth based on the presence of this plant today [171]. *Distichlis spicata* is a C_4_ plant and given its association with salty environments, it might be expected to possess relatively high *δ*^15^N values [172] but this has not been observed for northern Chile (*δ*^15^N = +4.6±3.1 ‰) [173] or northern Peru (−3.2 ‰) [111]. The similarity in both *δ*^13^C and *δ*^15^N values between the camelid fibers and the human hairs are consistent with both humans and camelids consuming a similar range of agricultural plants, suggesting that some animals were kept locally. An alternative possibility is that the non-highland camelid hair was derived from wild guanacos living at these lower altitudes. If, however, guanacos could consume a sufficient quantity of local plants, it begs the question as to why this would not have been equally plausible for llamas.

The absence of any non-highland isotopic compositions in the camelid bone collagen similar to that observed in the textiles may simply be a product of sample size, or it may imply that the primary motivation for keeping these animals locally was for fiber production rather than for meat or transport, underscoring the quintessential importance of textiles and camelid fiber in the Andean region [19, 174–176]. Niemeyer and Schiappacasse [52] estimated that given the low number of juvenile camelids identified in faunal assemblages, animal husbandry was oriented towards fiber production and/or transport, rather than for meat. However, they believed that the fiber was obtained from the adjacent *altiplano* because the environment near Huancarane would not be suitable for pastoral activities. They envisioned two strategies being employed by the local people: (1) possession of pastoral lands and herds in the highlands, and (2) the acquisition of livestock through exchange with llamas caravanners. These actions were also observed during the 1970s by these authors in this sector of the Camarones Valley. Our data suggest that by at least the LIP, groups living in the lower Camarones and Lluta Valleys kept small numbers of animals locally, but acquired the majority of their fiber through exchange with groups in the highlands. The reliance on imported fiber may have been driven by the limitations of the environment for camelid husbandry, necessitating an external supply.

## Conclusion

Our data suggest that the northern Chilean valleys were not able to support the same level of camelid husbandry as the northern valleys of Peru, but people were still attempting to raise small numbers of animals to produce fiber for textiles since at least the Late Intermediate Period. There was a significant shift in the isotopic compositions of camelid bone collagen between the LIP and the LH in the Lluta Valley, possibly because of alterations made to the distribution networks for camelids instituted by the Inca. These changes were not, however, visible in the textiles, which had comparable isotopic compositions between the LIP and LH.

## Supporting information

S1 Table Isotopic and elemental compositions for the bone collagen samples analyzed with contextual information. (XLSX)

S2 Table Isotopic and elemental compositions for the textile samples analyzed with contextual information. (XLSX)

S1 Appendix Supplementary methods. Relevant details on analytical uncertainty, quality assurance, and quality control.
(DOCX)

S1 File R scripts used in the analysis. (R)
