# Supplementary material for: Camelid husbandry in the Atacama Desert? A stable isotope study of camelid bone collagen and textiles from the Lluta and Camarones Valleys, northern Chile
Source: PLoS One. 2020 Mar 11;15(3):e0228332. doi: 10.1371/journal.pone.0228332 (PMC7065742; doi:10.1371/journal.pone.0228332)
Supplement: S1 Appendix — Relevant details on analytical uncertainty, quality assurance, and quality control. (DOCX) [file pone.0228332.s003.docx]

**S1 Appendix**

Camelid Husbandry in the Atacama Desert? A Stable Isotope Study of Camelid Bone Collagen and Textiles from the Lluta and Camarones Valleys, Northern Chile

**Stable Isotope Analysis − Calibration, Accuracy, and Precision**

Carbon and nitrogen isotopic and elemental compositions were determined using a Nu Horizon continuous flow isotope ratio mass spectrometer. Sample measurements were calibrated relative to VPDB (*δ*^13^C) and AIR (*δ*^15^N) using USGS40 and USGS66 or USGS41a (S3 Table [1, 2, 3]).

**S3 Table. Standard reference materials used for calibration of *δ*^13^C relative to VPDB and *δ*^15^N relative to AIR.**

| Standard | Material | Accepted *δ*^13^C  (‰, VPDB) | Accepted *δ*^15^N  (‰, AIR) |
| --- | --- | --- | --- |
| USGS40 | Glutamic Acid | −26.39±0.04 | −4.52±0.06 |
| USGS41a | Glutamic Acid | +36.55±0.08 | +47.55±0.15 |
| USGS66 | Glycine | −0.67±0.04 | +40.83±0.06 |

The following standards were used to monitor accuracy and precision (S4 Table). The isotopic compositions for the internal keratin standard represent long-term averages.

**S4 Table. Standard reference materials used to monitor internal accuracy and precision.**

| Standard | Material | Mean *δ*^13^C  (‰, VPDB) | Mean *δ*^15^N  (‰, AIR) |
| --- | --- | --- | --- |
| SRM−1 | Caribou bone collagen | −19.39±0.08 | +1.82±0.10 |
| SRM−2 | Walrus bone collagen | −14.81±0.06 | +15.60±0.11 |
| SRM−4 | Gluten | −26.76±0.08 | +5.25±0.11 |
| SRM−14 | Polar bear bone collagen | −13.67±0.07 | +21.60±0.15 |
| Casein | Casein | −26.98±0.13 | +5.94±0.08 |

S5 Table summarizes the mean and standard deviation of carbon and nitrogen isotopic compositions for all check standards, as well as the standard deviation for all calibration standards – the mean of the calibration standard for an individual run is predetermined to calibrate the data.

S5 Table. Mean and standard deviation of check and calibration standards for all analytical sessions containing data presented in this paper. Note that means for calibration standards are not presented as they are pre-determined to be equal to the known value.

| **Run ID** | **Standard** | **n** | ***δ*^13^C (‰, VPDB)** | | | ***δ*^15^N (‰, AIR)** | | |
| --- | --- | --- | --- | --- | --- | --- | --- | --- |
| 17-03 | USGS40 | 3 |  | ± | 0.02 |  | ± | 0.11 |
| 17-04 | USGS40 | 7 |  | ± | 0.08 |  | ± | 0.11 |
| 17-06a | USGS40 | 3 |  | ± | 0.03 |  | ± | 0.32 |
| 17-06b | USGS40 | 7 |  | ± | 0.05 |  | ± | 0.49 |
| 18-01 | USGS40 | 3 |  | ± | 0.04 |  | ± | 0.06 |
| 17-03 | USGS66 | 4 |  | ± | 0.06 |  | ± | 0.28 |
| 17-04 | USGS66 | 6 |  | ± | 0.07 |  | ± | 0.14 |
| 17-06a | USGS66 | 4 |  | ± | 0.08 |  | ± | 0.09 |
| 17-06b | USGS66 | 3 |  | ± | 0.04 |  | ± | 0.19 |
| 18-01 | USGS41a | 4 |  | ± | 0.05 |  | ± | 0.05 |
| 17-06b | Casein | 2 | −27.23 | ± | 0.02 | +6.13 | ± | 0.04 |
| 18-01 | SRM-1 | 4 | −19.34 | ± | 0.08 | +1.82 | ± | 0.10 |
| 17-03 | SRM-14 | 4 | −13.63 | ± | 0.07 | +21.50 | ± | 0.19 |
| 17-04 | SRM-14 | 4 | −13.67 | ± | 0.12 | +21.37 | ± | 0.04 |
| 17-06a | SRM-14 | 4 | −13.61 | ± | 0.06 | +21.47 | ± | 0.19 |
| 17-06b | SRM-14 | 2 | −13.60 | ± | 0.06 | +21.70 | ± | 0.09 |
| 18-01 | SRM-14 | 3 | −13.65 | ± | 0.04 | +21.50 | ± | 0.09 |
| 17-03 | SRM-2 | 4 | −14.76 | ± | 0.03 | +15.64 | ± | 0.23 |
| 17-04 | SRM-2 | 4 | −14.77 | ± | 0.07 | +15.41 | ± | 0.06 |
| 17-06a | SRM-2 | 4 | −14.76 | ± | 0.05 | +15.58 | ± | 0.05 |
| 17-06b | SRM-2 | 2 | −14.90 | ± | 0.01 | +15.43 | ± | 0.14 |
| 18-01 | SRM-2 | 4 | −14.68 | ± | 0.04 | +15.45 | ± | 0.13 |
| 17-03 | SRM-4 | 3 | −26.74 | ± | 0.03 | +5.32 | ± | 0.12 |
| 17-04 | SRM-4 | 2 | −26.81 | ± | 0.05 | +5.20 | ± | 0.14 |
| 17-06a | SRM-4 | 5 | −26.78 | ± | 0.04 | +5.30 | ± | 0.20 |

Nineteen samples were analyzed in duplicate. The pooled standard deviation for the duplicate pairs was ±0.19 ‰ for *δ*^13^C and 0.17 ‰ for *δ*^15^N.

**Analytical Uncertainty**

Standard uncertainty was calculated using the method present by Szpak et al. [4], which largely follows Magnusson et al.’s [5] approach. Standard uncertainty was determined to be ±0.19 ‰ for *δ*^13^C and ±0.28 ‰ for *δ*^15^N.

**Textile Sample Integrity**

For comparative purposes, we have reproduced the data from DeNiro’s [6] paper about collagen sample integrity (S1 and S2 Figs).


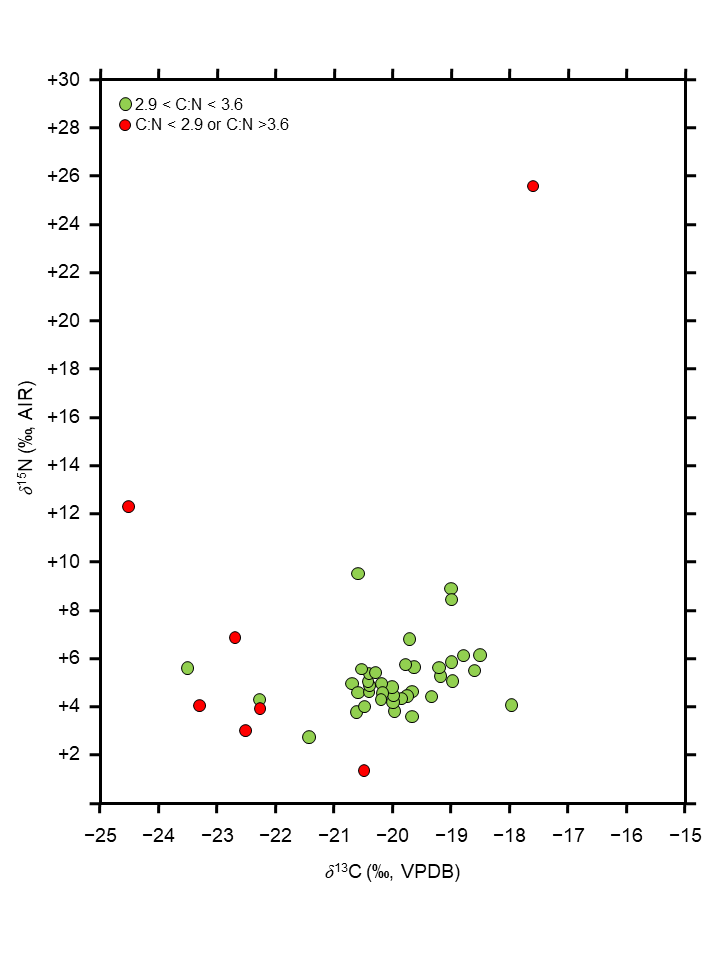


**S1 Figure**. Carbon and nitrogen isotopic compositions of bone collagen from ancient terrestrial herbivores (mammals). Samples are colored according to their atomic C:N ratios. Figure was produced using DeNiro’s data in Table 1 [6].

**
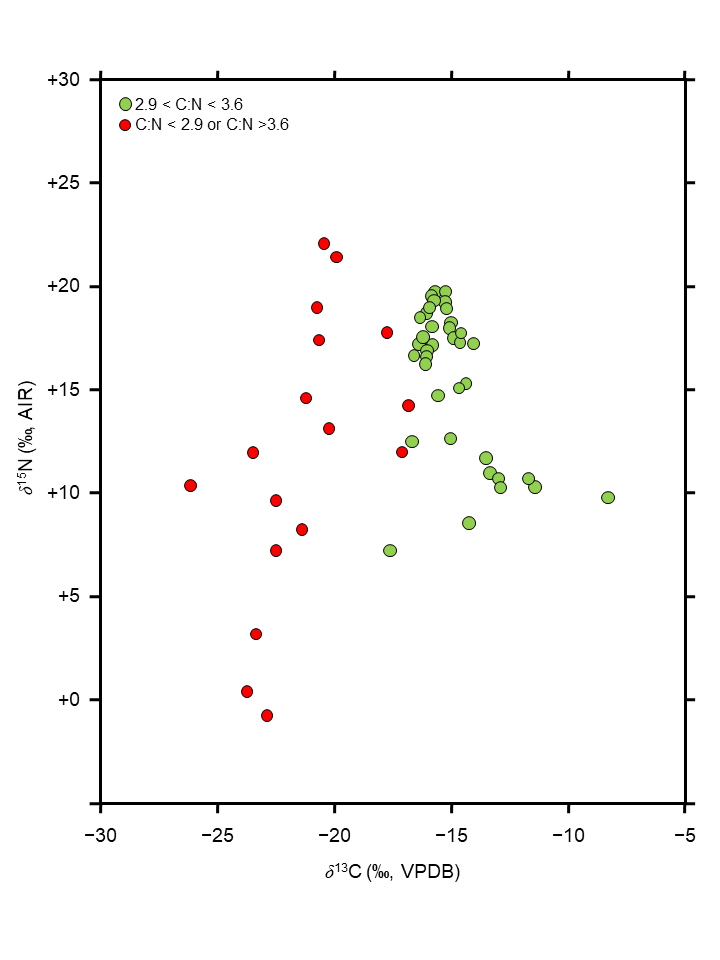
**

**S2 Figure**. Carbon and nitrogen isotopic compositions of bone collagen from ancient marine mammals. Samples are colored according to their atomic C:N ratios. Figure was produced using DeNiro’s data in Table 1 [6].

**References**

1. Qi H, Coplen TB, Geilmann H, Brand WA, Böhlke JK. Two new organic reference materials for δ^13^C and δ^15^N measurements and a new value for the δ^13^C of NBS 22 oil. Rapid Commun Mass Spectrom. 2003;17(22):2483-7. doi: 10.1002/rcm.1219.

2. Schimmelmann A, Qi H, Coplen TB, Brand WA, Fong J, Meier-Augenstein W, et al. Organic Reference Materials for Hydrogen, Carbon, and Nitrogen Stable Isotope-Ratio Measurements: Caffeines, n-Alkanes, Fatty Acid Methyl Esters, Glycines, l-Valines, Polyethylenes, and Oils. Anal Chem. 2016;88(8):4294-302. doi: 10.1021/acs.analchem.5b04392.

3. Qi H, Coplen TB, Mroczkowski SJ, Brand WA, Brandes L, Geilmann H, et al. A new organic reference material, l-glutamic acid, USGS41a, for *δ*^13^C and *δ*^15^N measurements − a replacement for USGS41. Rapid Commun Mass Spectrom. 2016;30(7):859-66. doi: 10.1002/rcm.7510.

4. Szpak P, Metcalfe JZ, Macdonald RA. Best Practices for Calibrating and Reporting Stable Isotope Measurements in Archaeology. J Archaeol Sci Rep. 2017;13:609-16. doi: 10.1016/j.jasrep.2017.05.007.

5. Magnusson B, Näykki T, Hovind Hv, Krysell M. Handbook for Calculation of Measurement Uncertainty in Environmental Laboratories. Nordtest Technical Report 537 ed. 3.1, 2012.

6. DeNiro MJ. Postmortem preservation and alteration of *in vivo* bone collagen isotope ratios in relation to palaeodietary reconstruction. Nature. 1985;317(6040):806-9. doi: 10.1038/317806a0.
